# Supplementary material for: Pathway crosstalk enables cells to interpret TGF-β duration
Source: NPJ Syst Biol Appl. 2018 May 28;4:18. doi: 10.1038/s41540-018-0060-5 (PMC5972147; doi:10.1038/s41540-018-0060-5)
Supplement: Supplementary file 1 — Supplementary figures [file 41540_2018_60_MOESM1_ESM.pdf]

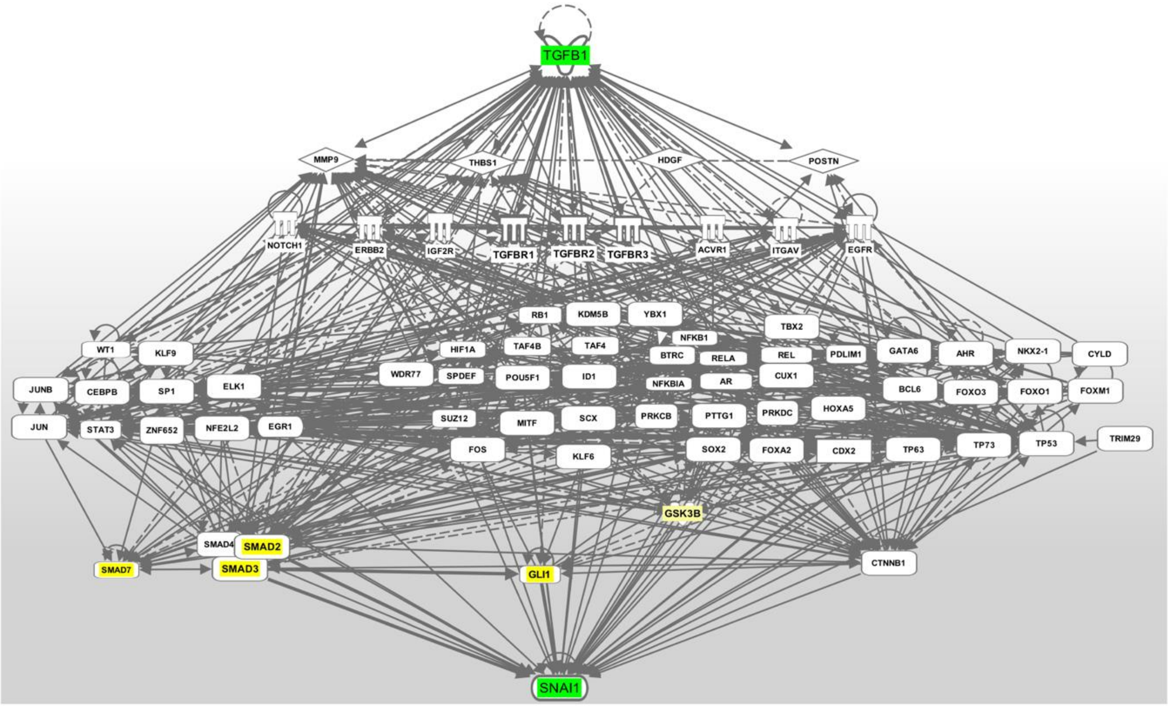

Figure S1 Network of TGF-β activating SNAIL1 reconstructed with IPA.

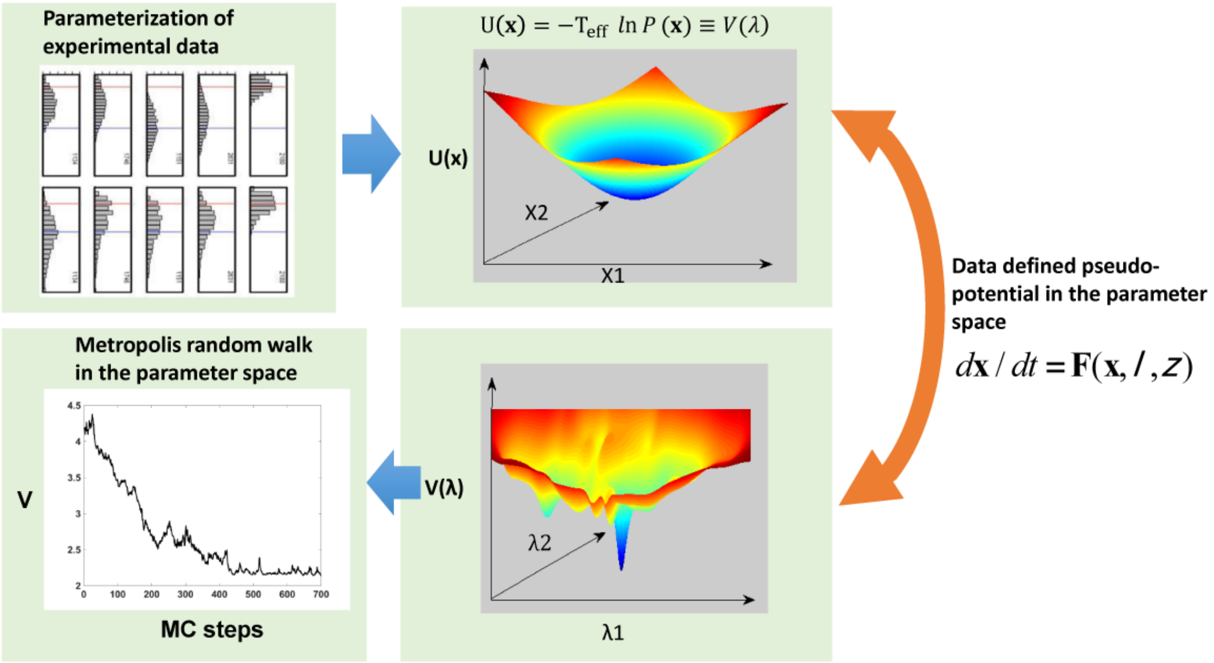

Figure S2 Schematic of the parameter space search approach. 1) Calculate single cell distributions of experimental observables. 2) Define pseudo-potentials from the parameterized distribution. 3) Obtain model parameter distributions that reproduce the distributions of experimental observables. (See parameter space searching in the SI for more detail).

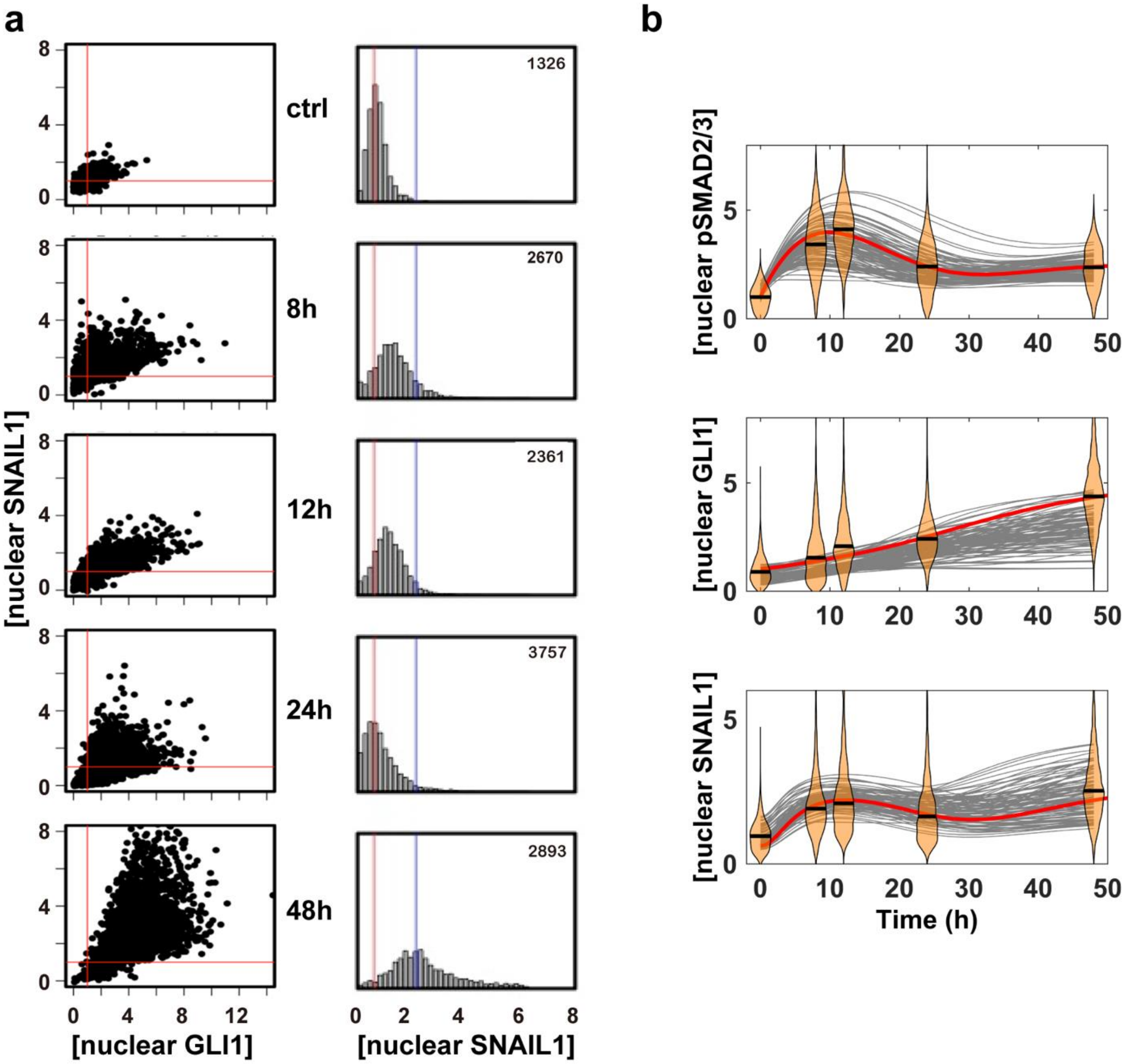

Figure S3 Supplemental results showing GLI1 contributes to the second wave of SNAIL1. (a) Scatted plot of measured nuclear GLI1 and SNAIL1 concentrations and the corresponding histogram representation for [nuclear SNAIL1]. The same sets of data of Fig. 3c are used. **(b)** The model of Fig. 2a with GLI1 reproduces the observed pSMAD2/3-SNAIL1 dynamics. To fit the SNAIL1 dynamics the exact temporal profile of GLI1 is not important except the requirement of its activation after 24 h.

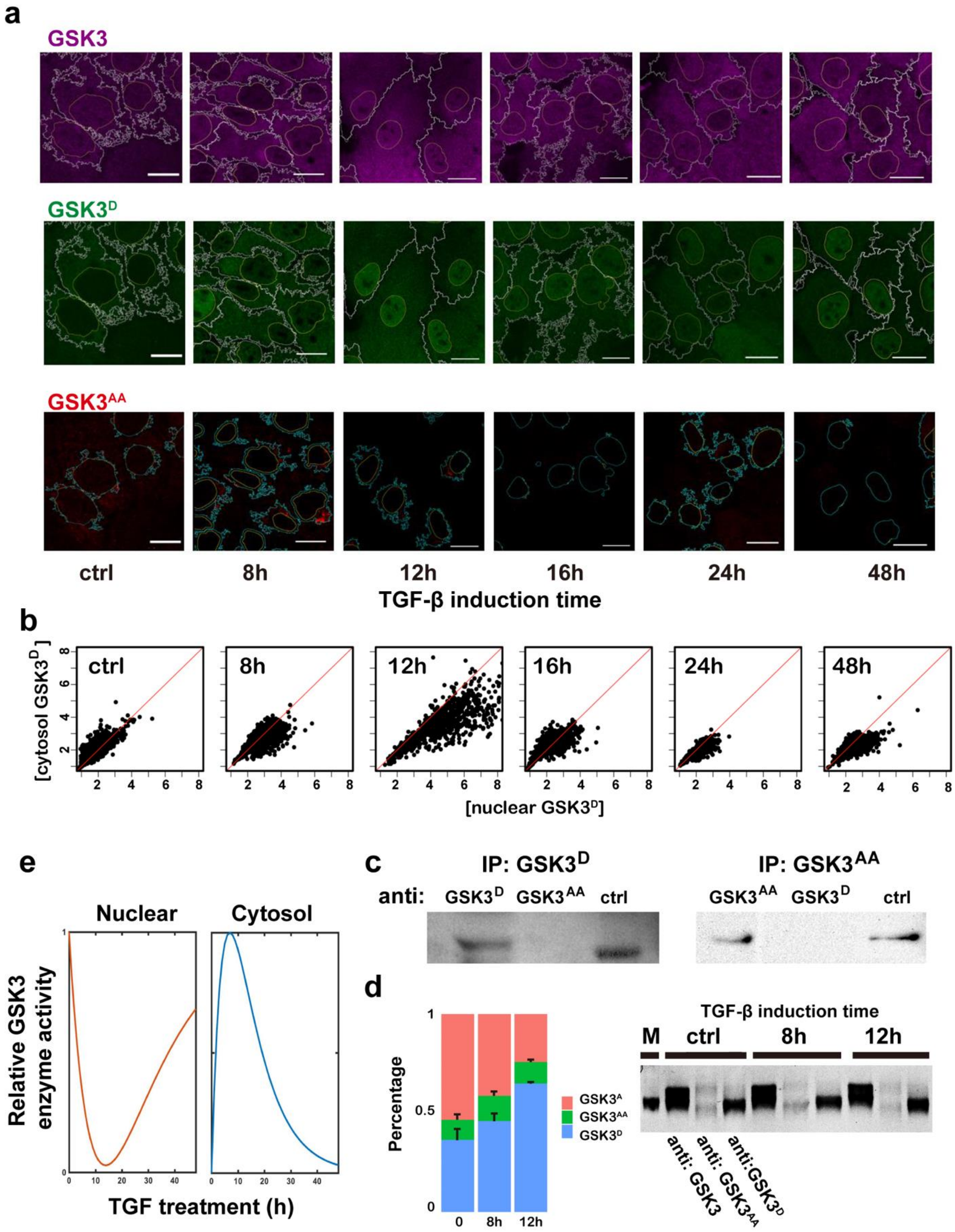

a
GSK3
GSK3D
GSK3AA
ctrl
8h
12h
16h
24h
48h
TGF-β induction time
b
[cytosol GSK3D]
[nuclear GSK3D]
ctrl
8h
12h
16h
24h
48h
e
Relative GSK3 enzyme activity
Nuclear
Cytosol
TGF treatment (h)
c
IP: GSK3D
IP: GSK3AA
anti:
GSK3D
GSK3AA
ctrl
GSK3AA
GSK3D
ctrl
d
Percentage
GSK3A
GSK3AA
GSK3D
0
8h
12h
TGF-β induction time
M
ctrl
8h
12h
anti: GSK3
anti: GSK3AA
anti:GSK3D

Figure S4 Supplemental results showing temporal switch between two phosphorylation forms of GSK3. (a) IF images showing the temporal switch between two phosphorylation forms of GSK3. **(b)** Scattered plots showing correlation between nuclear and cytosol concentrations of $GSK3^{D}$. **(c)** Immunoprecipitation studies showing two phosphorylation forms do not coexist. MCF10A cells were treated with TGF-β for 8 hours and the total proteins were harvested by RIPA. $GSK3^{D}$ and $GSK3^{AA}$ antibody were used for immunoprecipitation, respectively. $GSK3^{D}$ and $GSK3^{AA}$ proteins were used for western blot. Ctrl is sample that did not undergo immunoprecipitation. **(d)** Silver staining measurement of the relative amount of different GSK3 forms. The right figure shows a representative of three independent replicates. M refers to the marker with mass as 50 kd. **(e)** Relative GSK3 enzymatic activities in the cytosol and nucleus during TGF-β treatment.

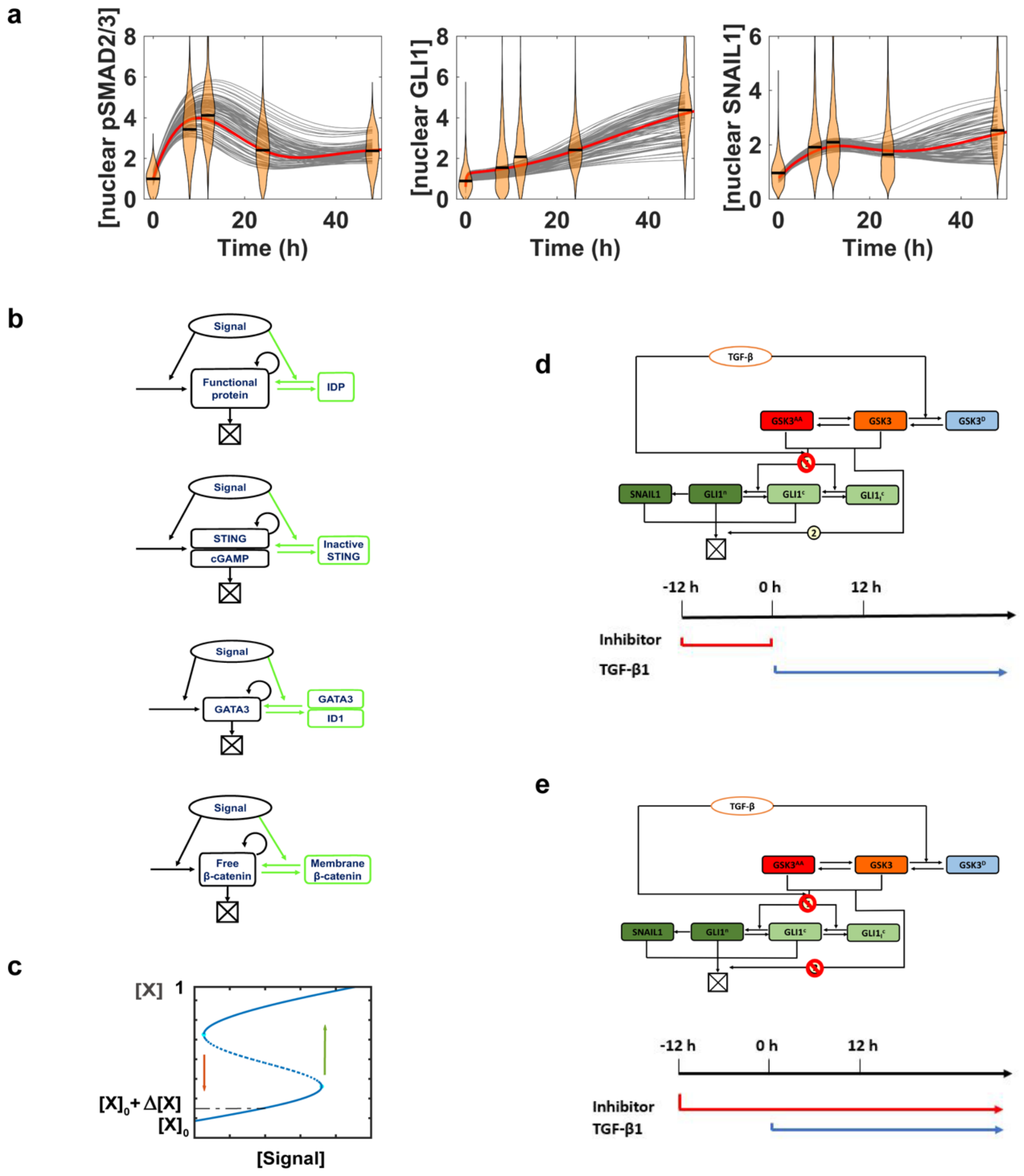

Figure S5. Supplemental results of the full model. (a) The model of Fig. 5a reproduces the observed GLI1 as well as pSMAD2/3-SNAIL1 dynamics. **(b)** Examples of regulatory factors having positive feedback loop and reservoir of molecules in inactive form that can be activated by another stimulus. IDPs refer to intrinsically disordered proteins, and some of them are transcription factors, which change into folded form and have higher DNA binding affinity upon binding of

cofactors or posttranslational modification. ID1 is a member of the family of inhibitors of DNA binding proteins. **(c)** Bifurcation diagram showing that the initial concentration boost is small compared to the concentration jump associated with external signal induced switch of cell states. **(d-e)** Schematics of the early and full GSK3 inhibition experiments.

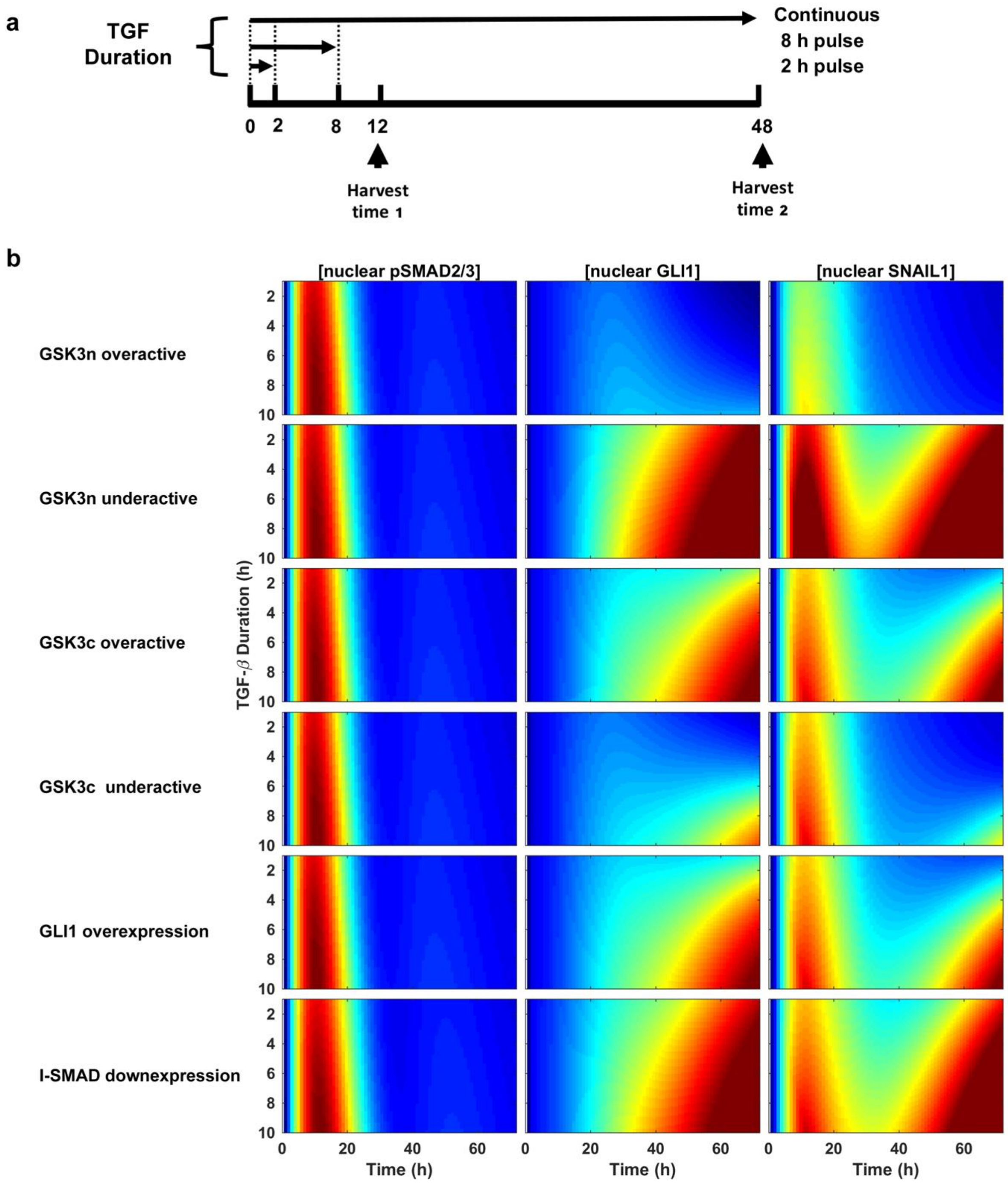
a
TGF
Duration
Continuous
8 h pulse
2 h pulse
0
2
8
12
48
Harvest
time 1
Harvest
time 2
b
[nuclear pSMAD2/3]
[nuclear GLI1]
[nuclear SNAIL1]
GSK3n overactive
GSK3n underactive
GSK3c overactive
GSK3c underactive
GLI1 overexpression
I-SMAD downexpression
TGF-β Duration (h)
Time (h)

c

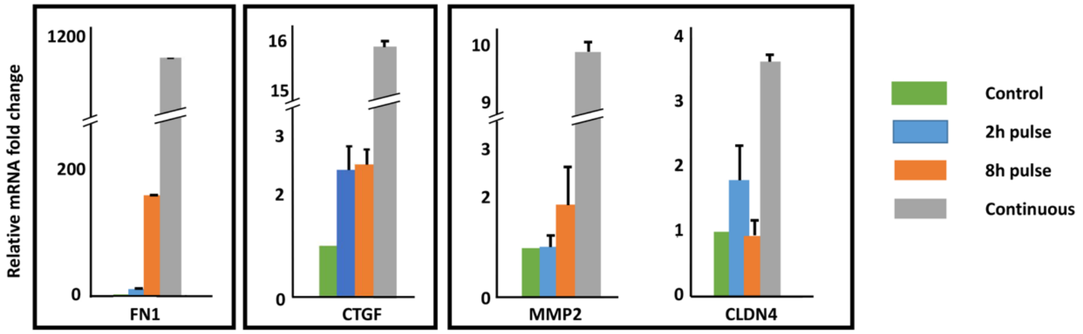

d

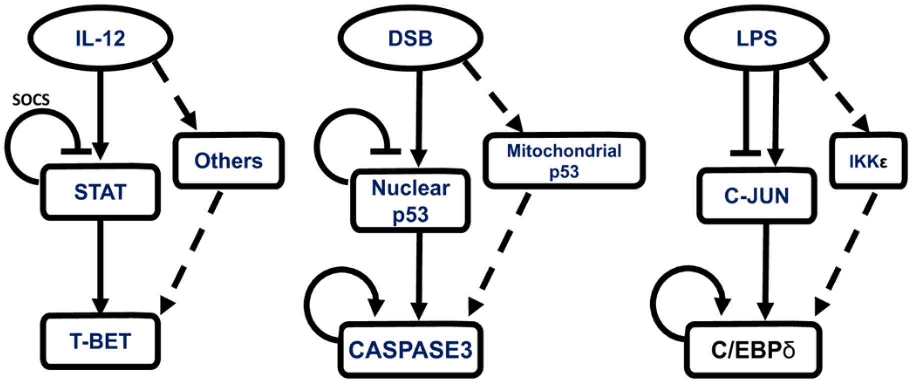

Figure S6 Supplemental results that cells can detect signal duration. **(a)** Schematic of TGF-β pulse experiments for fig. 6b and 6c. **(b)** Supplemental model results of pulsed TGF-β1 treatments with various mutations. **(c)** The mRNA levels of selected TGF-β activated genes at day 3 after different durations of TGF-β1 treatments. **(d)** Examples of other signaling transduction pathways that share similar motif structure as that of TGF-β signaling, including IL-12, DNA double strand breaking, and LPS.
