# Supplementary material for: Pathway crosstalk enables cells to interpret TGF-β duration
Source: NPJ Syst Biol Appl. 2018 May 28;4:18. doi: 10.1038/s41540-018-0060-5 (PMC5972147; doi:10.1038/s41540-018-0060-5)
Supplement: Supplementary file 2 — Supplementary information [file 41540_2018_60_MOESM2_ESM.docx]

# **Supplementary Materials**

**Mathematical modeling**

## Canonical TGF-β/pSMAD2/3/SNAILl pathway (Fig. 2a)

We used the following ordinary differential equation (ODE) model in Fig. 2 and Fig. S2.

***TGF-β/SMAD2/3 module.***

${[Smad]}^{'}=\left( kp_{smad0}+kp_{\mathrm{smad}}*TGF \right)*\frac{\mathrm{Sma}d_{\mathrm{all}}-\left[ \mathrm{Smad} \right]}{Jp_{smad0}+\left( \mathrm{Sma}d_{\mathrm{all}}-\left[ \mathrm{Smad} \right] \right)}\frac{1}{1+\frac{\mathrm{Sma}d_{I}}{\mathrm{Jd}p_{smad1}}} - dp_{\mathrm{smad}}*\frac{\left[ \mathrm{Smad} \right]}{\mathrm{Jd}p_{\mathrm{smad}}+\left[ \mathrm{Smad} \right]},$ (1)

$[Smad_{I}]'=k_{\mathrm{SmadI}}*[Smad]-\mathrm{kd}_{\mathrm{SmadI}}*[Smad_{I}]$, (2)

where [Smad] and$\left[ \mathrm{Sma}d_{I} \right]$ are the concentrations of pSMAD2/3 and inhibitory SMAD, respectively.

***SNAIL-miR-34 module.***

It is expanded from our previous model^34^ by considering transcription activation of SNAIL1 by pSMAD2/3 and TGF-β, and degradation of SNAIL1.

$\left[ \mathrm{snail} \right]_{n}^{'}= k0_{\mathrm{snail}}+k_{snail0}*\frac{[Smad]^{2}}{J_{snail0}^{2}+[Smad]^{2}}\frac{1}{1+\frac{\left[ \mathrm{SNAIL} \right]}{J_{snail2}}}-\mathrm{kd}_{\mathrm{snail}}*\left[ \mathrm{snail} \right]-\mathrm{kd}_{SR1}*[SR1]$ , (3)

$\left[ miR34 \right]_{n}^{'}= k0_{34}+\frac{k_{34}}{1 + \left( \frac{\mathrm{SNAIL}}{J1_{34}} \right)^{2}}- \mathrm{kd}_{34}*\left[ miR34 \right]-\left( 1-\lambda_{s} \right)*\mathrm{kd}_{SR1}*\left[ SR1 \right],$ (4)

$\left[ \mathrm{SNAIL} \right]^{'}= k_{\mathrm{SNAIL}}*\left[ \mathrm{snail} \right]-\mathrm{kd}_{\mathrm{SNAIL}}*\left[ \mathrm{SNAIL} \right],$ (5)

$\left[ miR34 \right]=\left[ miR34 \right]_{t}-\left[ SR1 \right],$ (6)

$\left[ \mathrm{snail} \right]=\left[ \mathrm{snail} \right]_{t}-\left[ SR1 \right],$ (7)

$\left[ SR1 \right]=Ks*\left[ \mathrm{snail} \right]*\left[ miR34 \right],$ (8)

where [$\mathrm{snail}$], $\left[ miR34 \right]$, $\left[ \mathrm{SNAIL} \right]$, $\left[ \mathrm{snail} \right], \left[ SR1 \right]$ are the concentrations of total *SNAIL1* mRNA, miR-34, SNAIL1 protein, free *SNAIL1* mRNA and miR-34-*SNAIL1* mRNA complex, respectively.

## Canonical TGF-β/SMAD/SNAILl pathway with GLI1 (Fig. 3, Fig. S3)

Taking into account the GLI1 self-activation and GLI1 mediated expression of *SNAIL1* mRNA, we added another ODE for GLI1 and revised the ODE of *SNAIL1* mRNA.

$\left[ \mathrm{GLI} \right]_{n}^{'}= k_{gli0}+k_{gli1}*\frac{[Smad]^{2}}{J_{gli1}^{2}+[Smad]^{2}} +k_{gli2}*\frac{{\left[ \mathrm{GLI} \right]_{n}}^{4}}{{\left[ \mathrm{GLI} \right]n}^{4}+J_{gli2}^{4}}-d_{\mathrm{gli}}*\left[ \mathrm{GLI} \right]_{n},$ (9)

${\left[ \mathrm{snail} \right]_{t}}^{'}= k0_{\mathrm{snail}}+\left( k_{snail0}*\frac{[Smad]^{2}}{J_{snail0}^{2}+[Smad]^{2}}+ k_{snail1}*\frac{{\left[ \mathrm{GLI} \right]_{n}}^{4}}{{\left[ \mathrm{GLI} \right]_{n}}^{4}+J_{snail1}^{4}} \right)\frac{1}{1+\frac{[SNAIL]}{J_{snail2}}}-\mathrm{kd}_{\mathrm{snail}}*\left[ \mathrm{snail} \right]-\mathrm{kd}_{SR1}*[SR1]$ , (10)

where $\left[ \mathrm{GLI} \right]_{n}$ is the concentration of nuclear GLI1. We used this ODE model to generate results in Fig. 3 and Fig. S3.

## Model for the GSK3/GLI module (Fig. 5a)

Since the process involves many steps and a detailed model would require many parameters to determine, instead we used two phenomenological time-dependent functions to qualitatively mimic the dynamics of the enzyme activities of cytosol GSK3 and nuclear GSK3 we experimentally measured (shown in Fig. 4b),

$A_{GSK}^{C}\left( t \right)=k_{\mathrm{GSKc}}*TGF*\left( 1-exp\left( -\frac{t}{a1} \right) \right)*exp\left( -\frac{t-b1}{a1} \right),$ (11)

$A_{GSK}^{n}\left( t \right)=1-k_{\mathrm{GSKn}}*TGF*\left( 1-exp\left( -\frac{t}{a2} \right) \right)*\left( \exp\left( -\frac{t-b2}{a2} \right) \right).$ (12)

Figure S4e shows the relative enzymatic activity. We played with different choices of the parameters, and found the results are insensitive to the choices provided there is an early pulsate increase of cytosol GSK3 enzymatic activity followed by a decrease of nuclear GSK3 enzymatic activity.

Furthermore, the basal pool of cytosol GLI1 is considered, which is by sequestered in the cytosol by SUFU but could translocate to the nuclear after SUFU is inactivated by the cytosol enzyme GSK3 activity. We used a revised ODE of nuclear GLI1 concentration derived with the quasi-equilibrium approximation (see below)

${\left[ \mathrm{GLI} \right]_{t}}^{'}\approx k_{gli0}+k_{gli1}*\frac{[Smad]^{2}}{J_{gli1}^{2}+[Smad]^{2}} + k_{gli2}*\frac{{\left[ \mathrm{GLI} \right]_{n}}^{4}}{{\left[ \mathrm{GLI} \right]_{n}}^{4}+J_{gli2}^{4}}-d_{\mathrm{gli}}*\left[ \mathrm{GLI} \right]_{n}*A_{GSK}^{n};$ (13)

***Derivation of GLI ODE***

We assumed the quasi-equilibrium approximation for the GLI nuclear and cytosol shuttling, the GSK3 regulated binding/unbinding between Sufu and GLI in the cytosol, and obtained the following equations,

${K2*\left[ \mathrm{GLI} \right]}_{c}*\left[ \mathrm{Sufu} \right]=(K1+A_{GSK}^{C})*[GLIsufu]$, (14)

$\left[ \mathrm{GLIsufu} \right]=\mathrm{Sufu}_{\max}-\left[ \mathrm{Sufu} \right]$ (15)

Thus we have

$\left[ \mathrm{GLIsufu} \right]=\mathrm{Sufu}_{\max}-(K1+A_{GSK}^{C})*\frac{\left[ \mathrm{GLIsufu} \right]}{K2*\left[ \mathrm{GLI} \right]_{c}}$ (16)

That is,

$\left[ \mathrm{GLIsufu} \right]=\frac{1}{1+\frac{(K1+A_{GSK}^{C})}{K2*\left[ \mathrm{GLI} \right]_{c}}}\mathrm{Sufu}_{\max}$ (17)

Also we have

$\left[ \mathrm{GLI} \right]_{c}=K3*\left[ \mathrm{GLI} \right]_{n}$, (18)

thus

$\left[ \mathrm{GLIsufu} \right]=\frac{{K3*K2*\left[ \mathrm{GLI} \right]}_{n}}{{K3*K2*\left[ \mathrm{GLI} \right]}_{n}+(K1+A_{GSK}^{C})}\mathrm{Sufu}_{\max}$ (19)

The total level of GLI1 is the sum of the three forms, $\mathrm{GLISufu}$, $\mathrm{GL}I_{c}$ and $\mathrm{GL}I_{n}$,

$\left[ \mathrm{GLI} \right]_{t}=\left[ \mathrm{GLI} \right]_{c}+\left[ \mathrm{GLI} \right]_{n}+\left[ \mathrm{GLIsufu} \right]=\left( K3*\left[ \mathrm{GLI} \right]_{n}+\left[ \mathrm{GLI} \right]_{n}+\frac{K2*K3*A_{GSK}^{n}}{{K3*K2*\left[ \mathrm{GLI} \right]}_{n}+(K1+A_{GSK}^{C})}\mathrm{Sufu}_{\max} \right)$. (20)

Thus, we obtained the relation among $\left[ GLI \right]_{n}$, $\left[ GLI \right]_{t}$ and $A_{GSK}^{C}$

$\left[ \mathrm{GLI} \right]_{n}$=$f\left( A_{GSK}^{C},\left[ \mathrm{GLI} \right]_{t} \right)$ , (21)

The total concentration of GLI1 is given by,

${\left[ \mathrm{GLI} \right]_{t}}^{'}= k_{gli0}+k_{gli1}*\frac{[Smad]^{2}}{J_{gli1}^{2}+\left[ \mathrm{Smad} \right]^{2}} + k_{gli2}*\frac{{\left[ \mathrm{GLI} \right]_{n}}^{4}}{{\left[ \mathrm{GLI} \right]_{n}}^{4}+J_{gli2}^{4}}-d_{\mathrm{gli}}*\left[ \mathrm{GLI} \right]_{c}*A_{GSK}^{C}-d_{\mathrm{gli}}*\left[ \mathrm{GLI} \right]_{n}*A_{GSK}^{n}.$ (22)

Given that our data shows that $\left[ \mathrm{GLI} \right]_{c}$is low throughout the process, we neglected the degradation term of $\left[ \mathrm{GLI} \right]_{c},$

${\left[ \mathrm{GLI} \right]_{t}}^{'}\approx k_{gli0}+k_{gli1}*\frac{[Smad]^{2}}{J_{gli1}^{2}+{[Smad]}^{2}} + k_{gli2}*\frac{{\left[ \mathrm{GLI} \right]_{n}}^{4}}{{\left[ \mathrm{GLI} \right]_{n}}^{4}+J_{gli2}^{4}}-d_{\mathrm{gli}}*\left[ \mathrm{GLI} \right]_{n}*A_{GSK}^{n}$ (23)

## *TGF-β pulse*

Since TGF-β1 can enter to cells through endocytosis, washing the extracellular TGF-β1 does not stop the signaling immediately. Therefore, we modeled the effective TGF-β1 concentration by the following equation,

$[TGF]\left( t \right)=\mathrm{TGF}_{0}*exp\left( -d_{\mathrm{tgf}}*\left( t-\mathrm{TGF}_{\mathrm{Duration}} \right)*Heaviside\left( t-\mathrm{TGF}_{\mathrm{Duration}} \right) \right).$ (24)

## *Full model*

By considering all the modules, the full model is as following,

${[Smad]}^{'}=\left( kp_{smad0}+kp_{\mathrm{smad}}*[TGF] \right)*\frac{\mathrm{Sma}d_{\mathrm{all}}-\left[ \mathrm{Smad} \right]}{Jp_{smad0}+\left( \mathrm{Sma}d_{\mathrm{all}}-\left[ \mathrm{Smad} \right] \right)}\frac{1}{1+\frac{[Smad_{I}]}{\mathrm{Jd}p_{smad1}}} - dp_{\mathrm{smad}}*\frac{\left[ \mathrm{Smad} \right]}{\mathrm{Jd}p_{\mathrm{smad}}+\left[ \mathrm{Smad} \right]},$ (25)

$\left[ \mathrm{Sma}d_{I} \right]^{'}=k_{\mathrm{SmadI}}*\left[ \mathrm{Smad} \right]-\mathrm{kd}_{\mathrm{SmadI}}*\left[ \mathrm{Sma}d_{I} \right],$ (26)

${\left[ \mathrm{GLI} \right]_{t}}^{'}= k_{gli0}+k_{gli1}*\frac{[Smad]^{2}}{J_{gli1}^{2}+[Smad]^{2}} + k_{gli2}*\frac{{\left[ \mathrm{GLI} \right]_{n}}^{4}}{{\left[ \mathrm{GLI} \right]_{n}}^{4}+J_{gli2}^{4}}-d_{\mathrm{gli}}*\left[ \mathrm{GLI} \right]_{c}*A_{GSK}^{C}-d_{\mathrm{gli}}*\left[ \mathrm{GLI} \right]_{n}*A_{GSK}^{n},$ (27)

${\left[ \mathrm{snail} \right]_{t}}^{'}= k0_{\mathrm{snail}}+\left( k_{snail0}*\frac{[Smad]^{2}}{J_{snail0}^{2}+[Smad]^{2}}+ k_{snail1}*\frac{{\left[ \mathrm{GLI} \right]_{n}}^{4}}{{\left[ \mathrm{GLI} \right]_{n}}^{4}+J_{snail1}^{4}} \right)\frac{1}{1+\frac{[SNAIL]}{J_{snail2}}}-\mathrm{kd}_{\mathrm{snail}}*\left[ \mathrm{snail} \right]-\mathrm{kd}_{SR1}*\left[ SR1 \right],$ (28)

$\left[ miR34 \right]_{n}^{'}= k0_{34}+\frac{k_{34}}{1 + \left( \frac{\mathrm{SNAIL}}{J1_{34}} \right)^{2}}- \mathrm{kd}_{34}*\left[ miR34 \right]-\left( 1-\lambda_{s} \right)*\mathrm{kd}_{SR1}*\left[ SR1 \right],$ (29)

$\left[ \mathrm{SNAIL} \right]^{'}= k_{\mathrm{SNAIL}}*\left[ \mathrm{snail} \right]-\mathrm{kd}_{\mathrm{SNAIL}}*\left[ \mathrm{SNAIL} \right]*A_{GSK}^{n},$ (30)

$A_{GSK}^{C}\left( t \right)=k_{\mathrm{GSKc}}*[TGF]*\left( 1-exp\left( -\frac{t}{a1} \right) \right)*exp\left( -\frac{t-b1}{a1} \right),$ (31)

$A_{GSK}^{n}\left( t \right)=1-k_{\mathrm{GSKn}}*[TGF]*\left( 1-exp\left( -\frac{t}{a2} \right) \right)*\left( \exp\left( -\frac{t-b2}{a2} \right) \right),$ (32)

$\left[ \mathrm{GLI} \right]_{n}=f([{GSK]}_{c},\left[ \mathrm{GLI} \right]_{t})$ (33)

$\left[ miR34 \right]=\left[ miR34 \right]_{t}-\left[ SR1 \right],$ (34)

$\left[ \mathrm{snail} \right]=\left[ \mathrm{snail} \right]_{t}-\left[ SR1 \right],$ (35)

$\left[ SR1 \right]=Ks*\left[ \mathrm{snail} \right]*\left[ miR34 \right].$ (36)

We used this ODE model to generate results in Fig. 6a and Supplementary Fig. S6b. In the above equations we chose a Hill coefficient of 2 for Smad and SNAIL1 based on their dimeric binding. We used a value of 4 for GLI1 for sufficient nonlinearity. To keep the model consistent, we used 4 in all our equations regard to GLI1.

## *Parameter space searching*

Step 1: Calculate single cell distributions of experimental observables. We calculated histograms of the distributions from the single cell experimental data. Suppose that we have *N* observables measured in *M* time points, we have an *N* × *M* dimensional distribution of the data. Since we used fixed cells and we had no information on the temporal correlation, we treated the distributions from different time points as independent, *i.e.*, $P=\prod_{i=1}^{M} P_{i}$.

Step 2: Define pseudo-potentials from the parameterized distribution. We defined a pseudo-scalar-potential function $U\left( \mathbf{x}_{1}, \mathbf{x}_{2}, .., \mathbf{x}_{M} \right)= -T_{eff} (lnP - lnP_{max}).$ The constant $T_{eff}$ is an effective temperature, which we chose $T_{eff}=1$. The constant term $lnPmax$ sets the potential to be zero at the peak position of the distribution, and does not affect the parameter space search results. This pseudo-potential is just an auxiliary scalar function for the following application of the Metropolis algorithm. If a mathematical model can faithfully describe the system dynamics, with given initial conditionals and non-adjustable parameter set of **ζ**, we should be able to find distributions of the parameter set **λ** (to take into account cell-to-cell heterogeneity), and generate the corresponding distributions of ($\mathbf{x}_{1}, \mathbf{x}_{2}, .., \mathbf{x}_{M}$) to reproduce *U.* That is, for a specific set of **λ**, $\mathbf{x}_{i} = \mathbf{x}_{i} (\mathbf{x}_{0};\boldsymbol{\lambda,} \boldsymbol{\zeta}), i = 1, \ldots M$, and $U(\mathbf{x}_{1}, \mathbf{x}_{2}, .., \mathbf{x}_{M}) \equiv V(\boldsymbol{\lambda})$. Unlike *U*, the function form of *V* can be very complex, but fortunately we do not need to know its explicit function form to perform the following Metropolis sampling.

Step 3: Obtain model parameter distributions that reproduce the distributions of experimental observables. Now it is clear why we define the pseudo-potential. We performed Monte Carlo random walks along the pseudo-potential *V* in the **λ** space using the Metropolis algorithm, just as how the algorithm is typically applied along real physical potentials. At each step with a set of **λ**, we generated a trial move **λ’ =** **λ + δλ.** We propagated the ODEs to obtain *V*(**λ**) and *V*(**λ’**), then use the Metropolis criteria to decide whether to accept the new move. If $V(\boldsymbol{\lambda}') \leq V(\boldsymbol{\lambda}) accept$ this step and update the parameter set $\boldsymbol{\lambda}= \boldsymbol{\lambda}\mathbf{'}$. If $V(\boldsymbol{\lambda}') >V(\boldsymbol{\lambda})$, accept this step with a probability $\exp(-\left( V\left( \boldsymbol{\lambda}^{'} \right)-V(\boldsymbol{\lambda}) \right)/T)$, with *T* = 1.

In our model, there is no feedback between the SMAD2/3 module and the SNAIL1/miR-34 module, thus we used a two-step to search the parameter space for the TGF-β/SMAD2/3 module,

1. Search the parameter space (nine parameters) in the SMAD2/3 module;
2. Search the parameter space (six parameters) for the SNAIL1/miR-34 module based on the 50 samples of good-fit parameter set of the SMAD2/3 module from step 1.

In step 2 some of the parameters in the SNAIL1/miR-34 module were fixed and used as a well-trained parameter set from our previous work ^34^. Instead only six new parameters that connect the module SMAD2/3 and module SNAIL1/miR-34 were considered in the parameter space searching.

When the GLI1 module was included, we again used the fact that there is no feedback between the SMAD2/3 module and the GLI1 module, and used a three-step searching procedure to reduce the computational efforts,

1. Search the parameter space (nine parameters) for the SMAD2/3 module;
2. Search the parameter space (seven parameters) for the GLI1 module;
3. Search the parameter space (six parameters) for the SNAIL1/miR-34 module based on the 50 samples of good-fit parameter set of the SMAD2/3 module the GLI1 module from step 1-2.

## Parameter change in various over-expression/down-expression or over-active/down-active conditions (Supplementary Fig. S6)

To produce the results in Fig. S6b, a 1.2-fold change of $k_{gli0}$ is used in the case of GLI1 over-expression, a 0.8-fold change of $k_{smadi}$ in the case of I-SMAD down-regulation. There is 0.8-fold change of $k_{gskn}$ in the case of over-active cytosol GSK3, 1.2-fold change of $k_{gskn}$in the case of under-active cytosol GSK3. Similarly, there is 1.2-fold change of $k_{gskc}$ in the case of over-active nuclear GSK3, and 0.5-fold change of $k_{gskc}$ in the case of under-active nuclear GSK3.

**Supplementary Movie S1: Subcellular localization of GSK3^AA^ (red).** Movies were composed from z-stack imaging.

**Supplementary Movie S2: Subcellular localization of GSK3^AA^ (red) overlaid with ERC (green) and DAPI (blue, nuclear area).** Movies were composed from z-stack imaging.
