# Supplementary material for: Pathway crosstalk enables cells to interpret TGF-β duration
Source: NPJ Syst Biol Appl. 2018 May 28;4:18. doi: 10.1038/s41540-018-0060-5 (PMC5972147; doi:10.1038/s41540-018-0060-5)
Supplement: Supplementary file 3 — Supplementary tables [file 41540_2018_60_MOESM3_ESM.docx]

# **Supplementary Table 1. Primer list**

| **Primers** | **Sequence** | **References** |
| --- | --- | --- |
| SNAIL1_QRT_F | ATCGGAAGCCTAACTACAGCGA | ^54^ |
| SNAIL1_QRT_R | CACGCCTGGCACTGGTACTTCT |  |
| GLI1_QRT_F | CTCCCTCGTAGCTTTCATCAAC |  |
| GLI1_QRT_R | GTGCTCGCTGTTGATGTGGTG |  |
| GAPDH_QRT_F | ACCACAGTCCATGCCATCAC | ^54^ |
| GAPDH_QRT_R | TCCACCACCCTGTTGCTGTA |  |
| FN1_QRT_F | AGCAGACCCAGCTTAGAGTT | ^55^ |
| FN1_QRT_R | GCAGAAGTGTTTGGGTGACT |  |
| CTGF_QRT_F | TTGGCCCAGACCCAACTA | ^56^ |
| CTGF_QRT_R | GCAGGAGGCGTTGTCATT |  |
| MMP2_QRT_F | tctcctgacattgaccttggc | ^57^ |
| MMP2_QRT_R | caaggtgctggctgagtagatc |  |
| CLDN4_QRT_F | ggctgctttgctgcaactgtc | ^58^ |
| CLDN4_QRT_R | gagccgtggcaccttacacg |  |

**Supplementary Table 2. The Parameters values of the best fit of the full model. The parameters shaded are searched with our algorithm illustration in Fig. S2 to fit the time course data of SMAD2/3 (Fig. 2C), SNAIL1 (Fig. 2C), GLI1 (Fig. 3C). The remaining parameters for the GSK3/GLI1 module are obtained through fitting the GSK3 temporal data (Fig. 4B). Other parameters for the SNAIL1/miR-34 module are taken from our previous model**^34^**. The initial condition is set as the steady state of model with no TGF-β.**

| **Parameter** | **Description** | **Value** |
| --- | --- | --- |
| **TGF-β/SMAD2/3 module** | | |
| $\mathrm{kp}_{smad0}$ | Basal activation rate of SMAD2/3 | 1.5778 μM/hr |
| $\mathrm{kp}_{\mathrm{smad}}$ | TGF-β dependent activation rate of SMAD2/3 | 0.2675 μM/hr |
| $\mathrm{Sma}d_{\mathrm{all}}$ | The total level of SMAD2/3 | 27.5377 μM |
| $\mathrm{dp}_{\mathrm{smad}}$ | Deactivation rate of SMAD2/3 | 1.4833 μM/hr |
| $\mathrm{Jp}_{smad0}$ | Michaelis constant of SMAD2/3 activation | 0.4198 μM |
| $\mathrm{Jdp}_{\mathrm{smad}}$ | Michaelis constant of SMAD2/3 deactivation | 0.3639 μM |
| $\mathrm{Jp}_{smad1}$ | Michaelis constant of SMAD-I mediated inhibition of SMAD2/3 activation | 0.8326 μM |
| $k_{\mathrm{smadi}}$ | Expression rate of inhibitory SMAD | 0.0254 μM/hr |
| $\mathrm{kd}_{\mathrm{smadi}}$ | Degradation rate of inhibitory SMAD | 0.0710 μM/hr |
| GSK3 /GLI1 module | | |
| $k_{\mathrm{GSKc}}$ | The activation rate of cytosol GSK3^AA^ enzyme activity | 1/hr |
| $k_{\mathrm{GSKn}}$ | The deactivation rate of nuclear GSK3 enzyme activity | 0.25/hr |
| a1 | Constant a of cytosol GSK3 enzyme activity | 10 |
| b1 | Constant b of cytosol GSK3 enzyme activity | 10 |
| a2 | Constant a of nuclear GSK3 enzyme activity | 20 |
| b2 | Constant b of nuclear GSK3 enzyme activity | 20 |
| $k_{gli0}$ | Basal transcription rate of *gli1* | 0.0003 μM/hr |
| $k_{gli1}$ | SMAD2/3-dependent transcription rate of *gli1* | 0.0453 μM/hr |
| $k_{gli2}$ | GLI1-dependent transcription rate of *gli1* | 0.2288 μM/hr |
| $d_{\mathrm{gli}}$ | Degradation rate of GLI1 | 0.0166 /hr |
| $J_{gli1}$ | Michaelis constant of SMAD2/3 -dependent transcription of *gli1* | 0.7563 μM |
| $J_{gli2}$ | Michaelis constant of GLI1-dependent transcription of *gli1* | 2.4192 μM |
| **SNAIL1/miR-34 module** | | |
| $k0_{\mathrm{snail}}$ | Basal transcription rate of *snail1* | 0.0034 μM/hr |
| $k_{snail0}$ | SMAD2/3-dependent transcription rate of *snail1* | 1.3942 μM/hr |
| $k_{snail1}$ | GLI1-dependent transcription rate of *snail1* | 43.5453 μM/hr |
| $J_{snail0}$ | Michaelis constant of SMAD2/3-dependent *snail1* transcription | 0.7522 μM |
| $J_{snail1}$ | Michaelis constant of GLI1-dependent *snail1* transcription | 7.2215 μM |
| $J_{snail1}$ | Michaelis constant of SNAIL1-dependent *snail1* transcription inhibition | 0.2012 μM |
| $\mathrm{kd}_{\mathrm{snail}}$ | Degradation rate of *SNAIL1* mRNA | 0.09 /hr |
| $\mathrm{kd}_{\mathrm{SR}}$ | Degradation rate of miR34-*SNAIL1* complex | 0.9 /hr |
| $k_{\mathrm{SNAIL}}$ | Translation rate of *SNAIL1* mRNA | 17 μM/hr |
| $\mathrm{kd}_{\mathrm{SNAIL}}$ | Degradation rate of SNAIL1 | 1.66 /hr |
| $k0_{34}$ | Basal production rate of miR-34 | 0.0012 μM/hr |
| $k_{34}$ | Production rate of miR-34 | 0.012 μM/hr |
| $J1_{34}$ | Michaelis constant of SNAIL1-dependent inhibition of miR-34 production | 0.15 μM |
| $\mathrm{kd}_{34}$ | Degradation rate of miR-34 | 0.035 /hr |
| $K_{s}$ | Affinity constant of miR-34 and *SNAIL1* mRNA | 100 /μM |
| $\lambda s$ | Recycle ratio of miR-34 | 0.5 |
